# Unravelling the pore network and gas dynamics in highly adaptive rubbery organic frameworks†

**DOI:** 10.1039/d2sc01355j

**Published:** 2022-04-13

**Authors:** Romain Dupuis, Mihail Barboiu, Guillaume Maurin

**Affiliations:** Institut Européen des Membranes, Adaptive Supramolecular Nanosystems Group, University of Montpellier, ENSCM-CNRS UMR5635, Place E. Bataillon CC047 34095 Montpellier France mihail-dumitru.barboiu@umontpellier.fr; ICGM, Univ. Montpellier, CNRS, ENSCM 34095 Montpellier France guillaume.maurin1@umontpellier.fr

## Abstract

Rubbery organic frameworks-ROFs have recently emerged as an intriguing class of dynamers by virtue of reversible connections between their building units. Their highly adaptative features at the origin of their spectacular self-healing properties made them also attractive candidates for the development of gas-selective membranes combining high selectivity and fast permeability. So far, little is known on the origin of this unique trait and this clearly hampers the exploitation of this class of dynamers in many areas where stimuli-responsive pore dynamics is of great importance. To address this lack of fundamental knowledge, herein we unravel the self-assembly process of ROFs *via* the development of an advanced computational methodology combining quantum and force field molecular simulations that enable the description of reversible connections of building units and the long-range organization of the cross-linked ROF network. We demonstrate that both accurate energy barriers associated with the covalent bond formation between the building units and presence of solvent are key parameters to ensure the *in silico* construction of reliable ROF structure models that are supported by a set of experimental data collected on synthesized ROFs including density, connectivity and porosity. Atomistic insights into the unusual guest-responsive pore dynamics of this intriguing class of dynamers are further gained with a special attention paid to the tunability of this pore flexibility by controlling the chemical composition of the building units. As a further stage, the dynamics of CO_2_ in these compliance frameworks is scrutinized to shed light on the mechanism at the origin of their promising performance as CO_2_-selective membranes. We highlight that guest-triggered pore dynamics enables the creation of a diffusion pathway to ensure effective gas transport throughout the whole ROF. This knowledge of the pore structure and its guest-responsive dynamics at the microscopic level is unprecedented in the field of dynamers and it is expected to pave the way towards the optimization of this class of adaptive porous frameworks for many potential applications. Interestingly, this computational approach can be transferable to the exploration of any complex disordered systems showing a high degree of flexibility and guest induced structure/pore reorganization.

## Abstract 2

Structure and gas transport molecular insight into the intriguing rubbery organic frameworks characterized by unique adaptative behavior owing to the reversible connections between their constitutive building units and their non-permanent porosity.

## Graphical abstract

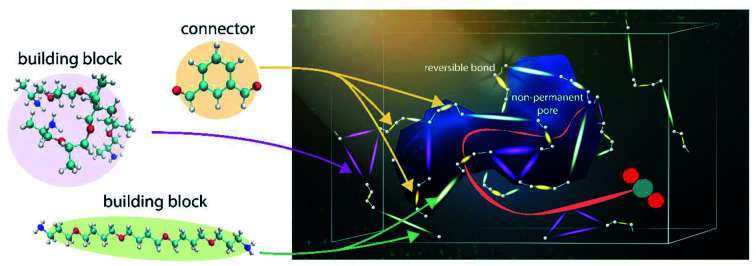

## 1 Introduction

Membrane technology is extensively used at the industrial level for a myriad of separation applications in liquid/gas phases as it offers many advantages, *e.g.* energy saving, simple design and easy scale-up.^1,2^ Membranes are mostly constructed from polymers^3–9^ while more recently various highly selective microporous materials including zeolites and metal organic frameworks MOFs have been processed as pure or composite membranes in association with glassy polymers to potentially address the threshold between permeability and selectivity for a range of gas mixtures.^10–15^

**Fig. 1:**
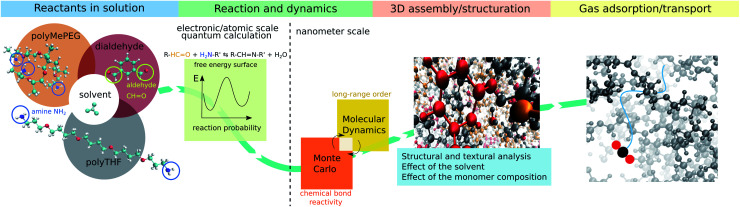
Overall workflow of the computational strategy to gain insight into the assembly of ROF dynamers and the resulting structure in the presence of solvent. The selected ROF/solvent system is composed of a connection center (isophthalaldehyde) and two amine-terminated connectors (stick-shaped polyTHF and star-shaped polyMePEG) with chloroform considered as a model solvent currently used for the synthesis of this ROF. (i) The free energy surface for the imine reaction is first determined using quantum calculations coupled with metadynamics. This enables the determination of the energy barrier for the assembly of the building units that is further implemented into a hybrid (ii) MC and (iii) MD scheme to deliver an atomistic ROF structure model further characterized in terms of porosity and connectivity prior to exploring (iv) its gas adsorption and transport properties.

Alternatively, rubbery organic frameworks (ROFs) constructed by reversibly connecting components under molecular control *via* a reversible chemical bond, can address the poor gas selectivity exhibited by classical rubbery polymeric membranes with a non-reversible covalent bond network, while maintaining high gas permeabilities.^16–18^ The high flexibility and mechanical compliance of this intriguing family of dynamic polymers named dynamers, confers excellent mechanical stability and both intrinsic and guest-responsive pore dynamics to the corresponding ROF membranes.^16–18^ These key features open new avenues towards the fabrication of adaptive membranes with high processability and optimal gas transport performances. Furthermore, the high chemical variability of the assembled building blocks of different shapes/sizes and chemical features enables custom-made design of almost infinite number of architectures, making this family of ROFs highly tunable for target gas separation.^19–22^ However, ROFs are poorly crystalline and often metastable most likely due to continuous reversible reorganization of their bond networks that is also at the origin of their unique self-healing or self-repair properties.^18^ The complexity and evolutionary nature of this family of dynamers explain that not only their formation mechanism but also their structure and guest-responsive structural changes are still unknown to date. Atomistic insight into the ROF structure organization is therefore required for a fine-tuning of this class of dynamers with the optimum pore network for target application. This challenging objective calls for the deployment of an innovative modelling strategy integrating (i) quantum calculations to accurately assess the energy barriers associated with the reactive assembly of building blocks and (ii) a hybrid Monte Carlo/molecular dynamics scheme that accounts for the reversible long-range order assembly of building blocks to deliver a reliable atomistic structure model of the dynamers. Herein, we devised such a computational strategy (workflow schematized in Fig. 1) that was applied to a prototypical dynameric ROF composed of a isophthalaldehyde core building block and two amine-terminated connectors, stick-shaped polytetrahydrofuran (polyTHF) and star-shaped glyceroltris(polypropylene glycol)ether (polyMePEG), which we previously demonstrated to act as an effective membrane for the selective CO_2_ capture.^16^ We unravel for the first time a ROF structure model showcased with the self-assembly of the building blocks mentioned above in the presence of solvent by explicitly considering the reversibility of the HC=N imine bond formation/dissociation, which connects the N atom of the amine group of polyTHF or of polyMePEG to the C atom of the aldehyde function belonging to isophthalaldehyde. The structure models built *in silico* were carefully analyzed in terms of local and long-range arrangements of the building units, connectivity degree and pore distribution/dimension. A set of experimental data collected on this ROF including the fractional free volume, density and assessment of the connectivity served as a preliminary validation of the structure models.

As a further stage, these structure models were used to predict the CO_2_ adsorption and transport properties of the ROFs. These Monte Carlo–Molecular dynamics simulations evidenced that these dynamers are highly adaptive and capable of adsorbing gas in evolutive emerging porosity owing to their reversible covalent bond network. Remarkably, our calculations revealed how the diffusion of CO_2_ can be modulated by changing the composition of the ROF in terms of polyTHF and polyMePEG concentrations, paving the way towards the selection of the best ROF composition to ensure optimum CO_2_ transport throughout the dynamers.

## 2 *In silico* construction of the atomistic ROF structure model

The first explored ROF system was made of 25% polyMePEG (M_*n*_ ∼ 3000 g mol^−1^) and 75% polyTHF (M_*n*_ ∼ 1100 g mol^−1^) connected *via* isophthalaldehyde centers (dialdehyde). Therefore, this dynamer denoted hereafter as S25, was modelled with the consideration of 150 diamine-terminated polyTHF building units and 50 triamine-terminated polyMePEG building units, corresponding to a total of 450 reactive sites (2 and 3 terminal functions for each polyTHF and polyMePEG respectively) distributed randomly with 225 isophthalaldehyde connectors in a simulation box of 50 × 50 × 50 Å^3^ dimension. Chloroform was considered as the standard solvent previously used for the synthesis of this ROF. This solvent was further removed by drying the membranes prior to testing their CO_2_ capture performance. The assembly of the dynamer was performed with a MC scheme to account for the imine bond formation/dissociation resulting from the reaction between the carbonyl moiety of isophthalaldehyde and the amine group of either polyTHF or polyMePEG (eqn (1)).

1 R–HC=O + H_2_N–R′ ⇔ R–HC=N–R′ + H_2_O

**Fig. 2:**
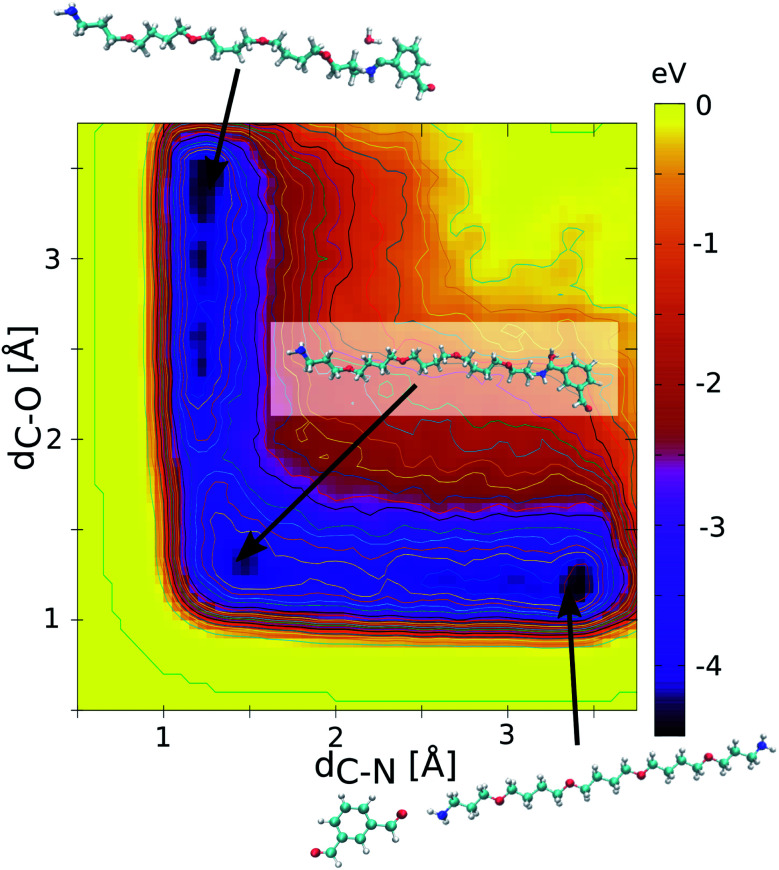
Free energy profile simulated by metadynamics using quantum calculations for the imine bond formation/dissociation resulting from the reaction between the carbonyl moiety of isophthalaldehyde and the amine group of polyTHF. The collective variables are the HC=N distance and the HC=O distance. The two minima are located at both ends of the reaction path. The preferred path is related to a dissociation of the HC=O bond to form a water molecule. The HC=N bond formation energy barrier (Δ*E*_f_) and bond dissociation (Δ*E*_d_) energy barrier are equal to 0.7 eV and 0.9 eV.

The MC algorithm first lists the pair of reactive sites along the MD trajectories, identified for new HC=N imine bond formation using a C–N distance criterion of 2.8 Å. The HC=N imine bond formation implies the release of one water molecule as shown in eqn (1). A cut-off of 2.5 Å for the distance between the C atom of the HC=N imine bond and the O atom of H_2_O is subsequently considered for the bond dissociation. The Metropolis acceptance criterion is then applied for the bond formation/dissociation with the following probability *P* = min(1, e^−*β*Δ*E*^), where Δ*E* is the energy barrier for either bond formation (Δ*E*_f_) or bond dissociation (Δ*E*_d_). The consideration of these energy barriers enables sampling of the equilibrium distribution of the dynamers. This critically calls for an accurate assessment of these corresponding energy barriers. To this purpose, quantum calculations were preliminarily conducted to determine the free energy profiles for the HC=N imine bond formation. All calculations were performed at the density functional theory (DFT) level using the PBE functional as implemented in the CPMD code^23^ and a cut-off energy of 90 Ry. A metadynamics scheme with gaussian-shaped biasing forces (width of 0.1 u.s. and height of 0.01 eV, frequency every 10 steps^24,25^) was selected to effectively scan the free energy profile of the reaction. In this case, two collective variables were considered: (i) the intermolecular C–N distance to enhance the imine bond formation and (ii) the intramolecular C–O distance in the dialdehyde to mimic the release of the O atom in the form of H_2_O during the reaction. Fig. 2 shows the calculated free energy profile of the HC=N imine bond formation for the 1 isophthalaldehyde/1 polyTHF pair introduced in a simulation box of 20 × 20 × 20 Å^3^ dimension. This profile exhibits three minima: one for the non-bonded polyTHF and dialdehyde building units, one for each assembled building unit implying a hemiacetal HOCH–NH bond or the HC=N imine bond just before and after releasing water respectively (see eqn (1)). Along this reaction path, the maximum energy barrier is 0.7 eV and 0.9 eV for the HC=N imine bond formation (Δ*E*_f_) and dissociation (Δ*E*_d_) respectively. Note that the CPMD calculations were performed without the inclusion of dispersion corrections. Since the energy barrier is rather high, the long-range dispersion energy contribution is expected to be negligible and therefore does not affect the energy difference between the energy barrier for the bond formation and bond dissociation. The free energy profile was equally calculated for the 1 isophthalaldehyde/1 polyMePEG and 1 isophthalaldehyde/2 polyTHF pairs leading to similar energy barriers (see Fig. S1 in the ESI† showing the energy profiles).

Once the bonds are equilibrated, the connectivity of the dynamer is modified accordingly and the structure is further relaxed using subsequent MD steps. All MD simulations are performed with the LAMMPS package^26^ using the GAFF force field^27^ for all atoms of the system treated as charged Lennard-Jones (LJ) sites. The atomic charges of the initial building units and chloroform molecules were extracted by the restrained electrostatic potential (RESP)^28^ charge fitting approach applied to the electrostatic potential calculated in Gaussian^29^ using the HF/6-31G* basis set.^27^ Moreover, when the components are connected, the charges of C and N atoms in the HC=N imine bond are modified using values calculated with the same RESP method computed on a system containing polyTHF bonded with isophthalaldehyde and a free water molecule (see ESI† for the values of charges in the bonded and non-bonded cases). The non-bonded contribution was treated as the sum of the LJ term with a cutoff of 10 Å and an electrostatic interaction calculated by means of the Ewald summation method as implemented in LAMMPS with a *k*-space value of 0.0001. Bonded-contribution includes harmonic potentials to model the stretching and bending modes, and cosine-based functions for dihedral and improper dihedral torsions (all the parameters are from the GAFF force field^27^). These MD simulations were performed in the NPT ensemble with a time step of 0.5 fs and using a Nose–Hoover barostat and thermostat with a relaxation time of 0.5 ps and 0.05 ps respectively. The building units are first equilibrated during 1 ns prior to starting the MC scheme and the MD simulations are further run for at least 50 ns. For every subsequent 1 ps MD simulation, one MC step is considered corresponding to 10 attempts to realize bond formation/dissociation according to the Metropolis criterion mentioned above (see movie of the bond association between polyTHF and isophthalaldehyde in the ESI†). For each attempt, the energy barrier of the HC=N bond formation/dissociation is considered to determine the probability of the reaction.

## 3 Analysis of the structural and textural features of the ROF model

**Fig. 3:**
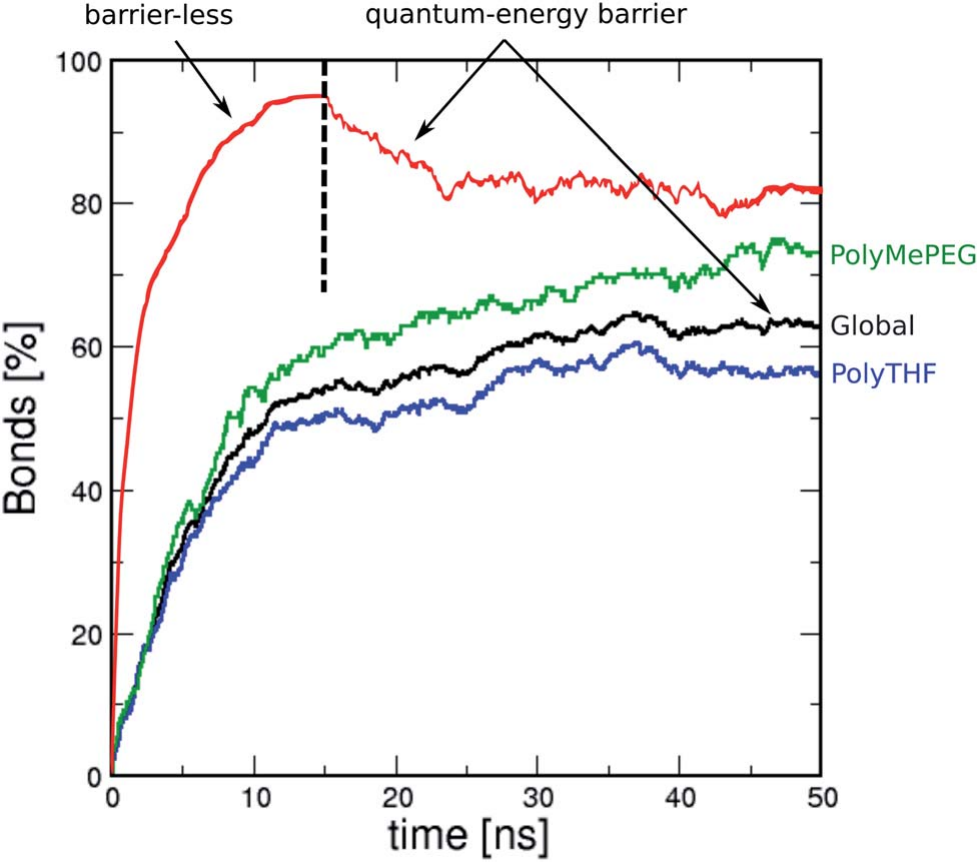
MD-time dependence of the averaged global bond connectivity (%) for the ROF S25 dynamer considering the energy barrier calculated by quantum calculations (Δ*E*_f_ = 0.7 eV and Δ*E*_d_ = 0.9 eV) (black). Comparison with the averaged bond connectivity calculated for polyTHF with dialdehyde (blue) and for polyMePEG with dialdehyde (green). The connectivity is also reported for the scenario considering barrier-less bond formation until 15 ns and for the energy barrier calculated by quantum calculations above 15 ns (red).

Fig. 3 shows the MD simulated time-dependence of the connectivity for the ROF S25 calculated as the fraction of carbonyl groups of isophthalaldehyde involved in the formation of a bond with polyTHF or polyMePEG. The connectivity converges to about 62% after 50 ns MD simulations (see the black line in Fig. 3) with isophthalaldehyde slightly more connected to polyMePEG (about 65%, green line) compared to polyTHF (about 57%, blue line). This evidences that the isophthalaldehyde units reacted at least once *via* their reactive carbonyl function, consistent with previous experimental findings.^16^

The density of the so-constructed model is 1.10 g m^−3^, which is in agreement with the experimental data previously reported for the synthesized ROF S25.^16^ We further assessed how the energy barrier for the bond formation/dissociation affects the connectivity of the structure model for ROF S25. The test case implies barrier-less bond formation and an infinite barrier for the bond dissociation, a typical scenario considered in most of the *in silico* polymerisation software (Fig. 3, red line). This leads to a very high connectivity (95%) reached after only 10 ns MD simulations. This resulting phase is so dense that the connectivity cannot be converted back to that of the initial simulated structure by again applying the energy barrier calculated at the DFT level (Fig. 3, black line). This observation emphasizes that an accurate evaluation of the energy barrier for the HC=N bond formation/dissociation is a key to achieve a reliable atomistic model for such dynamic ROF systems.

**Fig. 4:**
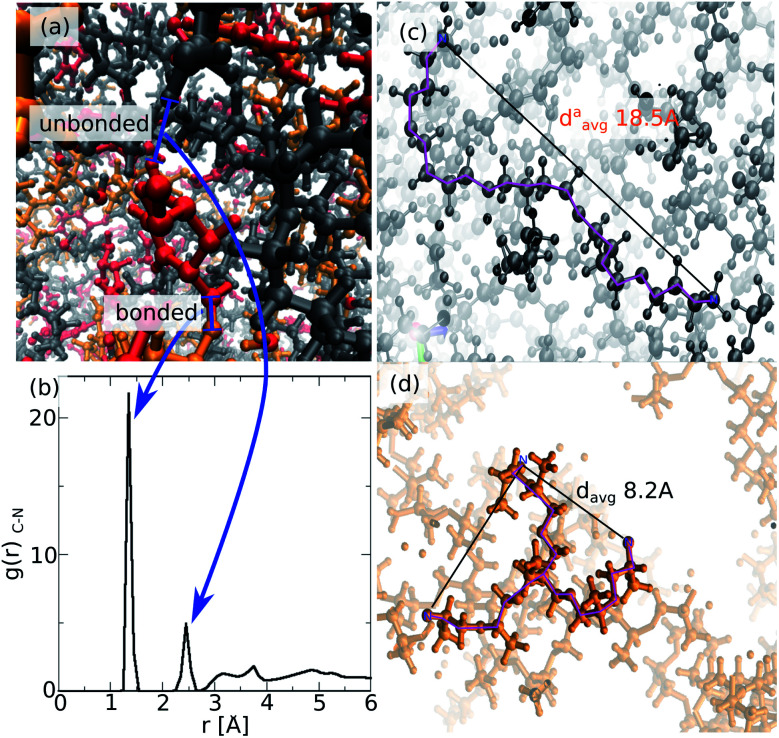
(a) Illustrative MD snapshot showing an isophthalaldehyde building unit (in red) bonded to polyMePEG (orange) throughout one aldehyde function while its second aldehyde function is free to connect to polyTHF (in gray). (b) Radial distribution function calculated for the C–N pair over the MD run. (c) Illustrative MD snapshot of polyTHF (highlighted in purple) embedded in the ROF and its corresponding end to end distance. (d) Illustrative MD snapshot of polyMePEG embedded in the ROF and its corresponding end to end distance. In both figures (c and d) other building units have been omitted for clarity.

The resulting atomistic model of ROF S25 is highly disordered as illustrated in Fig. 4a with intertwined chains that still contain non-bonded amine and aldehyde reactive sites consistent with incomplete connectivity as reported in Fig. 3. This is reflected in the radial distribution function (RDF) calculated for the C–N pair (Fig. 4b) that exhibits the first peak at 1.5 Å assigned to the intramolecular HC=N bond length and the second one at 2.5 Å accompanied by additional contributions above 3 Å corresponding to non-bonded distances between unreacted amine and dialdehyde groups. Fig. 4a also shows that the connections of the dialdehyde building units can be achieved *via* one polyTHF and one polyMePEG molecules. The length of the polyTHF chain in the ROF S25 (about 18 Å) is reduced by about 10 Å in average compared to its length exhibited in the gas phase (about 28 Å), due to the angular coiling of the molecule in the ROF (Fig. 4c). This strongly suggests that the polyTHF building units are able to re-extend if an external stimulus is applied to the dynamer leading to a high flexibility of this family of soft solids. On the opposite, the star-shaped polyMePEG building units (see Fig. 4d), are only slightly shrunk (*d*_avg_ = 8.2 Å) compared to their conformation in the gas phase (*d*_avg_ = 8.5 Å).

**Fig. 5:**
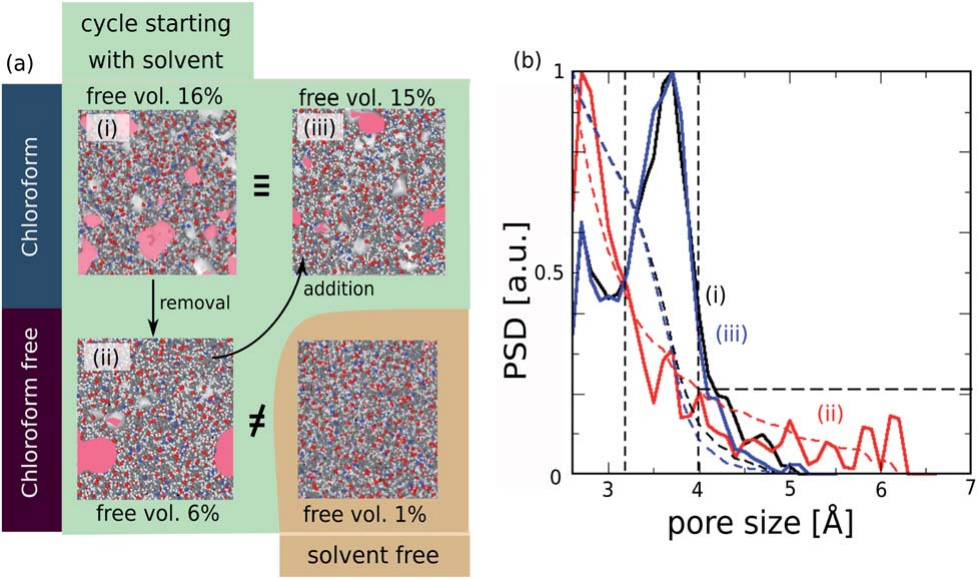
(a) Evolution of the fractional free volume of the ROF S25 depending on the conditions used in the MD simulations for the generation of the structural models. The section in green corresponds to a cycle starting with the structure constructed with the inclusion of the solvent (configuration (i)) followed by the subsequent removal (configuration (ii)) and reincorporation (configuration (iii)) of the solvent. The pores accessible to a probe molecule of 3.3 Å (kinetic diameter of CO_2_) are represented in pink. The non-porous structure generated without considering chloroform initially is illustrated in gold. (b) Pore size distribution (solid lines) and contribution to the total fractional free volume pore density (dashed lines) for the structures (configuration (i), black), (configuration (ii), red) and (configuration (iii), blue).

The resulting ROF S25 structure model shows a fractional free volume of 16% (Fig. 5a – configuration (i)) calculated with a probe molecule of 3.3 Å, corresponding to the pore regions accessible to CO_2_ considered as a model molecule diffusing in the corresponding membrane.^16^ This fractional free volume is in line with the corresponding experimental data reported previously for this ROF (about 11–12%).^16^ Analysis of the pore size distribution (PSD) reported in Fig. 5b shows that the pores of ROF S25 exhibit a predominant contribution at 3.8 Å.

When the chloroform is released from the ROF S25 structure model, the MD simulations converge towards a denser phase as revealed by a much lower fractional free volume (6%) (Fig. 5a – configuration (ii)). This reduction of porosity is associated with a substantial decrease of the population of pores with a dimension of 3.8 Å that are shifted to small voids below 3 Å as illustrated in the corresponding PSD plot (Fig. 5b). Remarkably, the re-incorporation of the solvent in this guest-free model leads to re-opening of the porosity as observed in Fig. 5a – configuration (iii), with an associated fractional pore volume (15%) and PSD profile (Fig. 5b) that fit with the corresponding data obtained for the model constructed initially in the presence of chloroform. These overall computational findings deliver an unprecedented atomistic insight into the responsive pore dynamics of the ROFs upon guest adsorption/desorption. This prediction is also in line with the unique self-healing properties of this family of dynamers previously revealed.^18^ Note that experimentally the self-healing mechanism occurs within minutes after two cut pieces are held together.

We equally envisaged the construction of an atomistic ROF structure model without the initial presence of solvent. While the resulting solvent-free structure (Fig. 5a – configuration (iv)) shows connectivity very similar to that of the structure model built with chloroform (see Fig. S2 in ESI†), it is almost non-porous (fractional free volume lower than 1%) and much denser than the structure obtained after solvent removal (Fig. 5a – configuration (ii)). This conclusion emphasizes that the solvent is a key parameter to account for the modelling of highly flexible dynamers. It is to be noted that the consideration of solvent is only rarely considered in the modelling of structure models for standard polymeric systems and therefore can be a severe drawback for the most complex polymers implying a high degree of flexibility. In our specific case, chloroform is expected to facilitate the mobility of the building units in the simulation box in order to increase the probability of the reactive groups to be close to each other for initiating the HC=N bond formation. This is evidenced by a faster convergence of the constructed structure model in the presence of solvent (see connectivity in Fig. S2†). In addition, chloroform as a non-polar solvent interacts with the organic part of the chains and directs the structural organization of the dynamer that affect its pore distribution and hence its resulting fractional pore volume.

## 4 Prediction of the CO_2_ transport in the ROF

**Fig. 6:**
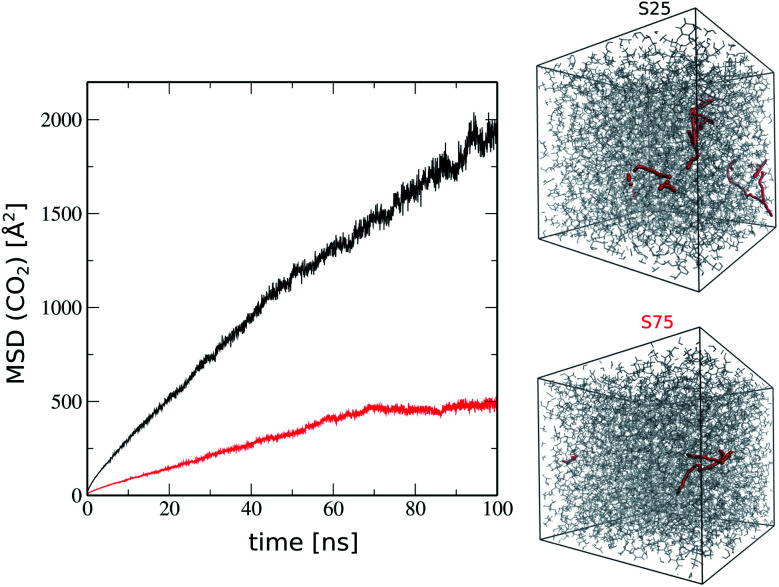
Mean square displacement (MSD) calculated for CO_2_ in ROF S25 (black) and ROF S75 (red) over 100 ns MD simulation runs at 300 K. The snapshots on the right show the diffusion path followed by one tagged CO_2_ molecule in each ROF. The self-diffusion coefficient (*D*_s_) of CO_2_ in ROF S25 is calculated using the Einstein relation applied in the 8–10 ns range.

As a further stage, we explored the CO_2_ transport in this generated ROF S25 structure model to gain unprecedented microscopic insight into the attractiveness of this family of dynamer membranes for CO_2_ separation. First, Monte Carlo simulations using the CADSS software^30^ were performed to load CO_2_ molecules on the ROF S25 structure obtained after removing chloroform and further MD simulations were performed for 50 ns at room temperature to explore the guest diffusion (100 CO_2_ molecules were adsorbed at 3 bar considered as a typical pressure). The simulated time-dependent mean square displacement (MSD) profile of the CO_2_ molecule, shown in Fig. 6 exhibits initially a ballistic regime and further adopts a linear trend characteristic of the Fickian-diffusion regime. The self-diffusion coefficient (*D*_s_) of CO_2_ calculated using the Einstein relationship was found to be 5 × 10^−11^ m^2^ s^−1^ at 300 K (see Fig. 6) in excellent agreement with the previous experimental work on the corresponding membrane that estimated a diffusion coefficient of about 10^−11^ m^2^ s^−1^.^16^ For comparison we investigated the diffusion of CO_2_ in a ROF structure model denoted as ROF S75 containing 75% of polyMePEG corresponding to 50 polyTHF building units, 150 polyMePEG building units and 275 dialdehyde building units introduced in the simulation box of the same size as that of ROF S25 (50 × 50 × 50 Å^3^). This ROF S75 shows a connectivity of 70%, which is slightly higher than that of ROF S25 (65%) while the fractional free volume of 12% is a bit lower (*vs.* 16% for ROF S25), consistent with a slight increase of the density from 1.08 to 1.10 g m^−3^. In order to have a direct comparison with ROF S25, the same number of CO_2_ molecules was added to the ROF S75 model. Fig. 6 shows that the mobility of CO_2_ in this ROF S75 is rather limited and did not enable us to extract a self-diffusion coefficient. This prediction again is in excellent agreement with the experimental observation previously reported showing that a higher concentration of polyMePEG incorporated in the ROF membrane leads to a drop of the CO_2_ diffusion. Interestingly the ratio of the MSD values for CO_2_ between ROF S25 and ROF S75 after 100 ns is about 4 : 1, in qualitative agreement with the experimental measurements revealing a ratio of permeability of 5 : 1 for the two corresponding membranes.^16^

We further explored the microscopic origin of the distinct gas transport behavior exhibited by ROF S25 and ROF S75. After adsorption, the pore size distributions of ROF S25 and ROF S75 (see Fig. S4†) and their free volumes (10% and 9% respectively) are similar. However, ROF S25 shows a higher degree of flexibility as reflected by its lower calculated bulk modulus (2.4 GPa) as compared to ROF S75 (4.9 GPa) (see ESI† for details of the calculations). This trend is consistent with a larger concentration of the flexible polyTHF molecule present in ROF S25. Therefore the higher CO_2_ permeability observed for ROF S25 stems from its higher flexibility that enables to generate CO_2_-triggered adaptive paths for optimum CO_2_ transport throughout the whole system.

## 5 Conclusion

Herein, we unraveled the first structural model for the family of dynameric rubbery organic frameworks *via* the development of a computational methodology integrating quantum-and force field molecular simulations to account for the reactivity of the assembly of building units and the long-range organization of the cross-linked network. The *in silico* constructed atomistic models were validated with experimental data collected on the synthesized ROFs in terms of porosity, connectivity and density. We demonstrated that the consideration of an accurate energy barrier associated with the chemical assembly of the building units *via* covalent bonding and the presence of solvent, both features most often neglected in the standard assembly software, is a key to achieve reliable structure models for this family of highly adaptive frameworks. Remarkably, their pore networks were revealed to be reversibly guest-responsive featuring preferential pathways for the guest molecules to diffuse at the origin of the very attractive CO_2_ permeability performance of the corresponding membranes. This flexible behaviour was shown to depend on the chemical composition of the frameworks controlled by the concentration of the different building units considered initially for the assembly process. This knowledge is expected to pave the way towards the optimization of the pore structure/dimension of this class of adaptive porous frameworks to tune their gas transport properties and guide the fabrication of membranes with improved performance for the separation of molecules of industrial importance. Beyond this class of dynamers, the advanced computational methodology developed in this study is transferable to the exploration of any complex disordered systems showing a high degree of flexibility and guest-induced structure/pore reorganization.

## Data availability

All the computational details and simulated extra data are provided in the ESI.†

## Author contributions

Conceptualization: RD, MB & GM. Methodology development and computational analysis: RD under the supervision of GM. Writing – original draft: RD & GM. Writing – review and editing: all authors.

## Conflicts of interest

There are no conflicts to declare.

## Supplementary Material

SC-013-D2SC01355J-s001
