# Supplementary material for: Unravelling the pore network and gas dynamics in highly adaptive rubbery organic frameworks
Source: Chem Sci. 2022 Apr 13;13(18):5141–7. doi: 10.1039/d2sc01355j (PMC9093169; doi:10.1039/d2sc01355j)
Supplement: SC-013-D2SC01355J-s001 [file SC-013-D2SC01355J-s001.pdf]

Electronic Supplementary Material (ESI) for Chemical Science.
This journal is © The Royal Society of Chemistry 2022

Supplemetary materials - Unravelling pore network and gas dynamics in highly adaptive rubbery organic frameworks

Romain Dupuis,[†,‡] Mihail Barboiu,[*,†] and Guillaume Maurin[*,‡]

†*Institut Europeen des Membranes, Adaptive Supramolecular Nanosystems Group, University of Montpellier, ENSCM-CNRS, UMR5635, Place E. Bataillon CC047, 34095 Montpellier, France*

‡*Institut Charles Gerhardt Montpellier, University of Montpellier, CNRS, ENSCM, Montpellier, France.*

E-mail: mihail-dumitru.barboiu@umontpellier.fr; guillaume.maurin1@umontpellier.fr

Atomic partial charges for the building blocks of ROFs

Dialdehyde

Atom	q	x	y	z
c	0.0337	1.498132507	2.4456631778	22.3210150984
c	0.0337	1.4981776805	2.1392957333	19.9143640286
c	-0.241	1.4969874318	1.6055187532	21.2052955477
c	-0.241	1.4993213989	3.8356224079	22.1367617468
c	-0.241	1.4997749562	3.530791663	19.7429037659
c	-0.241	1.4998880609	4.3721551075	20.8520704368
c	0.501	1.4989639555	1.858431709	23.6781060609
c	0.501	1.4985838528	1.2286510879	18.7491239606
o	-0.501	1.5039740269	2.5008758887	24.7161756404
o	-0.501	1.5049920388	1.587587062	17.5822245489
h	0.234	1.4951673087	0.5205994505	21.3426126502
h	0.244	1.5000399956	4.4762211336	23.0186693106
h	0.08	1.5011854253	3.9286158041	18.7277965323
h	0.18	1.495728022	5.3804361349	20.8278739026
h	-0.004	1.4944353362	0.7401294658	23.696725139
h	-0.004	1.4922431754	0.1415371243	19.0127125695

polyTHF

Atom	q	x	y	z
c	0.125	1.5428341992	3.0854612692	10.3400250545
c	0.032	1.324683673	2.1848378428	11.5418432758
c	0.39	1.4957963945	2.9243118032	12.8638656641
c	0.34	1.5483309601	3.0800483227	7.9645344876
c	-0.02	1.5462359441	2.1340956344	6.7771375721
c	-0.02	1.5325436562	2.8746865393	5.4425652863
c	0.324	1.5822993857	1.9336509842	4.2527833381
c	0.282	1.5728574532	1.9151514749	1.876926952
c	-0.005	1.5243140192	2.8395827792	0.6717550319
c	0.004	1.5880844919	2.0844326403	-0.6558166896
c	0.275	1.5300072756	3.0124897969	-1.8579394663
c	0.327	1.496069035	3.0404569919	-4.2270286148
c	0.003	1.5289735519	2.1420310254	-5.4515922281
c	-0.077	1.4519758024	2.9234985779	-6.7643991118
c	0.373	1.4998438553	2.0054757825	-7.9727852566
c	0.089	1.491825768	1.9706607755	-10.3340082203
c	0.0742	1.4244356322	2.8538760508	-11.5670598675
c	0.358	1.5132683011	2.047189523	-12.8596748498
o	-0.494	1.3999327163	2.3152052954	9.1540473418
o	-0.518	1.5358113588	2.7057135273	3.0590032496
o	-0.519	1.5801400812	2.2385425084	-3.0526319006
o	-0.493	1.4175539366	2.7787678318	-9.1643737986
n	-1.038	1.4707621783	1.9820950983	13.9853395572
n	-1.055	1.3978057402	2.927131959	-14.0256450869
h	0.389	1.5959110633	2.4638908952	14.8752247577
h	0.360	0.5748730436	1.4944804553	14.0314157726
h	-0.051	2.4724624628	3.4333599746	12.8786459019
h	-0.051	0.7283171817	3.7211754627	12.9336537511
h	0.0236	2.0317909164	1.3438758714	11.5076102581
h	0.0236	0.3121616602	1.7541646495	11.4861555555
h	0.010	2.5560298432	3.5352228824	10.3800382685
h	0.010	0.8150908242	3.9220871448	10.3369376014
h	-0.029	2.4934915034	3.6589276271	7.99501861
h	-0.029	0.7210434978	3.8141250736	7.8784916294
h	0.008	0.6652169289	1.4800192694	6.8608835516
h	0.008	2.4301990755	1.4816288347	6.8445176637
h	0.006	2.3849235421	3.5681029686	5.3794058359
h	0.006	0.6222565917	3.4873089908	5.3611092595
h	-0.028	0.7270833125	1.2286166379	4.2826103181
h	-0.028	2.5084757386	1.3239700373	4.2789742634
h	-0.020	0.7154835615	1.2122711994	1.8655302643
h	-0.020	2.4970804762	1.3025071691	1.857642656
h	0.008	2.3611107446	3.5509393749	0.748158697
h	0.008	0.6000183783	3.4349406873	0.7274631662
h	0.006	0.7535048427	1.3703493103	-0.724767622
h	-0.028	2.3385636827	3.7607103282	-4.2509137806
h	-0.028	0.5598846117	3.634289664	-4.2089977183
h	0.001	0.6923007676	1.4293781391	-5.3858365126
h	0.001	2.4538811163	1.54529538	-5.4233951563
h	0.018	2.2851952991	3.6390562112	-6.8325976599
h	0.018	0.5247351349	3.5146649227	-6.8064365752
h	-0.030	0.6619029065	1.2806896549	-7.9370503739
h	-0.030	2.4385040319	1.4152411932	-7.9706242553
h	0.010	0.6583334753	1.2388354733	-10.33765894
h	0.010	2.4353489917	1.3873111989	-10.3267913337
h	0.020	2.2509883454	3.5818480432	-11.5265627359
h	0.020	0.489603325	3.4323976593	-11.565709897
h	-0.038	0.6777933252	1.3299597457	-12.8973736269
h	-0.038	2.4438578775	1.443912062	-12.849065592
h	0.393	1.4289885517	2.3952416581	-14.895416348
h	0.370	2.1712548156	3.5933299678	-14.0540559002
h	-0.038	2.4438578775	1.443912062	-12.849065592
h	0.393	1.4289885517	2.3952416581	-14.895416348
h	0.369	2.1712548156	3.5933299678	-14.0540559002

PolyMePEG

Atom	q	x	y	z
c	-0.232	6.33504	9.23363	12.8126
c	-0.067	6.66275	8.98456	11.3044
c	-0.018	6.15891	7.58839	10.8358
c	0.125	4.67324	7.67103	10.7147
c	0.401	6.70601	7.29891	9.46311
c	0.096	6.60863	6.5319	11.8585
c	-0.092	8.78376	5.50529	11.6575
c	0.329	10.2959	5.80496	11.6991
c	-0.430	10.3189	7.32581	11.3066
c	-0.091	12.4938	4.58153	11.7063
c	0.618	13.5176	3.75641	10.892
c	-0.678	14.6957	3.56735	11.9104
c	-0.044	4.23672	5.42553	9.66585
c	-0.122	6.95294	8.3981	7.21617
c	0.496	3.00648	4.44985	9.43295
c	0.479	8.40431	8.35628	6.68661
c	-0.408	7.98322	8.68552	5.21231
c	-0.278	1.78951	5.44569	9.38774
c	-0.0624	2.2715	2.92611	7.55273
c	0.734	2.74181	2.0157	6.36471
c	-0.500	4.02044	2.75163	5.82416
c	-0.072	10.6364	7.29214	7.21015
c	0.653	11.2948	5.87322	7.42741
c	-0.508	12.8129	5.97959	7.96415
o	-0.316	8.03452	6.76382	12.0591
o	-0.455	4.05772	6.30534	10.8411
o	-0.481	6.63732	8.54321	8.63528
o	-0.470	11.1199	4.80216	11.0106
o	-0.524	3.34887	3.62421	8.26861
o	-0.477	9.16318	7.10166	6.93891
n	-0.982	12.8777	2.50575	10.268
n	-0.974	10.48	4.9334	8.2383
n	-1.157	1.67605	1.65975	5.36566
h	0.056	6.25723	9.8048	10.6775
h	0.0516	7.7999	8.91446	11.2144
h	0.045	6.07498	6.54312	12.8502
h	0.045	6.44339	5.51135	11.4809
h	0.095	8.54698	5.18719	10.6374
h	0.095	8.41888	4.68677	12.3031
h	0.036	10.6627	5.75954	12.7567
h	0.118	11.3628	7.78912	11.2984
h	0.118	9.76684	7.5462	10.3549
h	0.118	9.80479	7.84354	12.1347
h	0.079	12.2489	4.01557	12.6982
h	0.079	12.8989	5.58496	12.0214
h	0.017	13.8014	4.34636	9.97694
h	0.167	15.6147	3.03446	11.4276
h	0.167	15.1727	4.52308	12.311
h	0.167	14.4456	2.86015	12.761
h	0.387	11.9158	2.69596	9.71237
h	0.320	12.76	1.66797	11.1126
h	0.064	4.41554	8.21264	9.841
h	0.064	4.33275	8.19346	11.7006
h	0.104	5.16714	4.76641	9.7128
h	0.104	4.27113	6.05275	8.73343
h	-0.047	2.83754	3.64987	10.2233
h	0.066	0.821524	4.85407	9.24718
h	0.066	1.8202	6.04754	10.3704
h	0.066	1.85343	6.16965	8.54652
h	0.121	4.83881	2.95614	6.52363
h	0.121	4.39662	2.18457	4.93567
h	0.121	3.66932	3.83332	5.55446
h	0.116	13.4798	5.14719	7.58607
h	0.116	13.3146	6.91506	7.62906
h	0.116	12.7142	5.96235	9.15796
h	0.096	7.12088	8.01899	4.94629
h	0.096	7.6741	9.7556	5.05578
h	0.096	8.78707	8.51579	4.49395
h	0.064	1.45155	3.60917	7.10226
h	0.064	1.57601	2.32565	8.25809
h	-0.089	2.98329	0.986976	6.72099
h	0.348	0.625952	1.57419	5.78489
h	0.424	1.80625	0.813563	4.62914
h	-0.051	6.1872	6.4268	8.92691
h	-0.051	7.78087	6.96667	9.55107
h	0.115	6.36534	9.23011	6.72874
h	0.115	6.4895	7.45784	6.79368
h	0.013	9.06278	9.2538	7.04776
h	0.047	11.1385	7.84987	6.3784
h	0.047	10.834	7.92306	8.05976
h	-0.040	11.2985	5.43273	6.40538
h	0.301	9.443	5.17186	7.81276
h	0.374	10.4716	5.06357	9.37223
h	0.061	5.25119	8.89656	13.0796
h	0.061	7.07083	8.59568	13.3963
h	0.061	6.49223	10.3007	13.1278

Atomic partial charges for the ROFs when imine bond is formed

In order to take into account the charge transfer during the imine bond reaction, we computed the charges on the system when the bond is formed between polyTHF and the dialdehyde molecule. Three atoms have a charge that is strongly modified when the bond is formed: the O atom that initially belongs to the dialdehyde (q = -0.50) and finally belongs to the water molecule (q = -0.71); the C atom that initially belongs to the dialdehyde (q = 0.51) and finally belongs to the CH=N imine bond (q = -0.66); and the N atom that initially belongs to polyTHF (q = -1.0) and finally belongs to the CH=N imine bond (q = .41). The total charge for these three atoms is equal to -0.99 before the reaction and equal to -0.96 after the reaction. In order to maintain the charge constant in the system, when the bond is formed, the charge for O in the water molecule was taken as equal to -0.74. Considering that all the imine bonds in the system are equivalent, the charges are updated in the MC algorithm as follows: $qO_{\text{bonded}} = qO_{\text{non-bonded}} - 0.24$; $qC_{\text{bonded}} = qC_{\text{non-bonded}} - 1.17$; and $qN_{\text{bonded}} = qN_{\text{non-bonded}} + 1.41$.

Free energy surface for the imine bond formation/dissociation

In the dynameric systems, each dialdehyde can connect to one or two polymers (eiher polyTHF or polyMePEG). The free energy surface was calculated using Metadynamics at the DFT level for the different cases (see Fig. S. 1). No significant differences are observed in the value of the energy barrier for all the different cases, which is in agreement with the fact that the reaction and the local chemical environment is similar for forming/dissociating the imine bond in each of the cases. In all cases, the reaction happens with a shortening of the C-N distance and a water molecule (or an OH group) is released after the imine bond is formed.

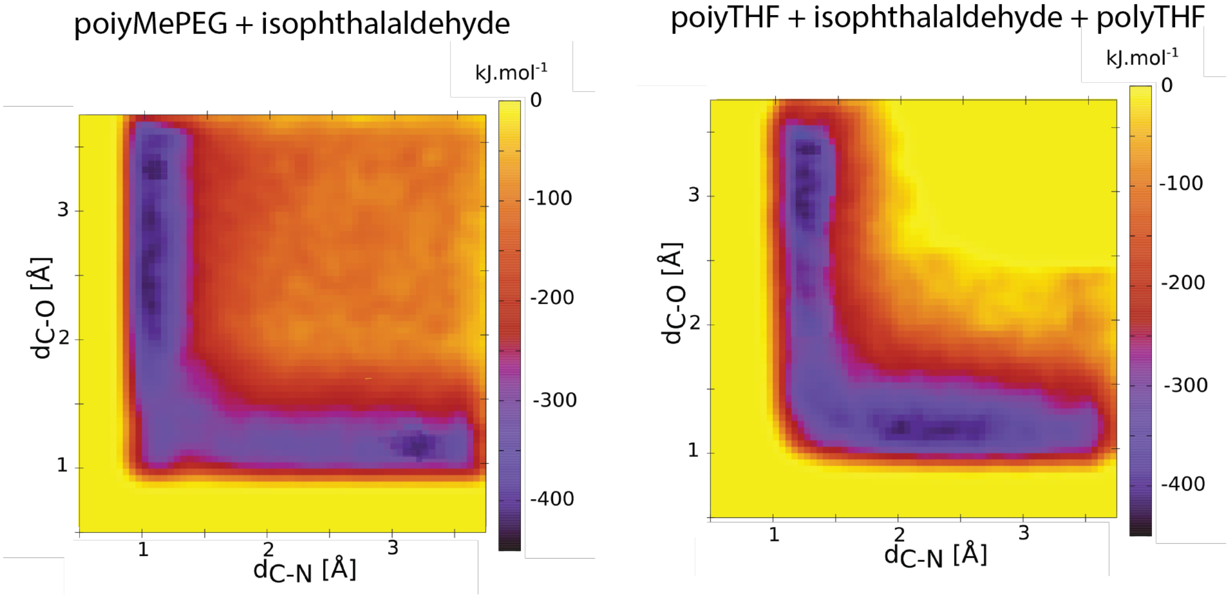

Fig. S. 1: Energy surface calculated for the imine bond in three different molecule association (polyMePEG + isophthalaldehyde / polyTHF + isophthalaldehyde + polyTHF).

Connectivity of the ROF

Fig. S. 2 shows that a system with no solvent reaches a connectivity slightly lower than the same system containing chloroform. Moreover, the increase in connectivity is slower for the system with no solvent, which reflects the effect of solvent on the diffusion of species in the system.

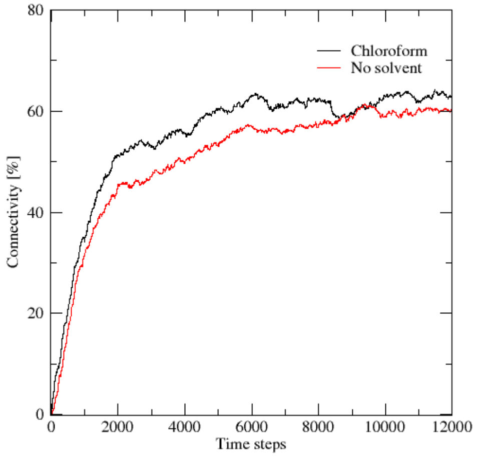

Fig. S. 2: MD-time dependence of the averaged global bond connectivity (%) for the ROF S25 dynamer considering the energy barrier calculated by quantum calculations in the presence and absence of solvent

Bulk modulus

The bulk modulus were estimated for S25 and S75 have been estimated by calculating the variation of the volume against applied pressure for three different pressures 0 GPa, 0.1 Gpa, and 1 Gpa. The calculated bulk modulus is 2.4 GPa for S25 and 4.9 GPa for S75.

Radial distribution function

The radial distribution functions, showing the structure of polyTHF, obtained by quantum calculations and classical calculations are shown in Fig. S 3. The distribution functions are similar with both approaches.

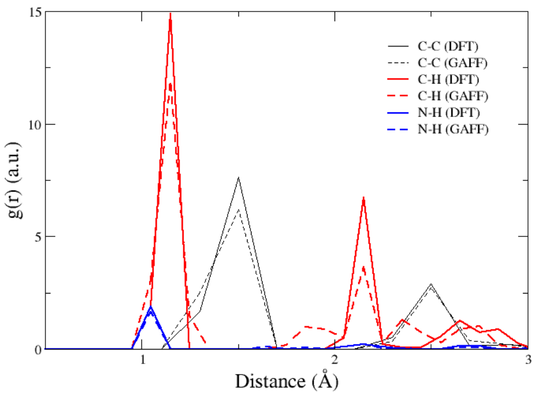

Fig. S. 3: Radial distribution functions computed with CPMD (DFT calculation) and LAMMPS using the GAFF force field performed on a single configuration after relaxation of the geometry of polyTHF.

Pore size distribution after gas adsorption

The pore size distribution after gas adsorption is shown in Fig. S 4. In both systems, there is a peak at 3.3 Å, which corresponds to the kinetic diameter of CO_2.

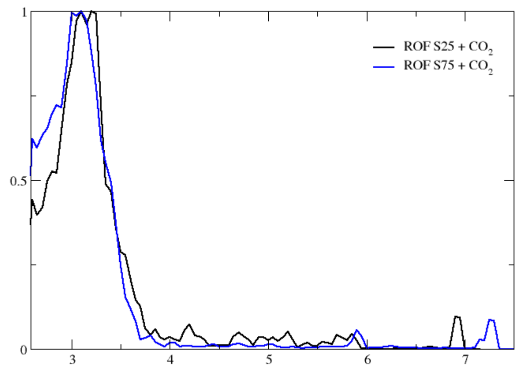

Fig. S. 4: Pore size distribution for ROF S25 and ROF S75 after CO_2 adsorption (CO_2 molecules are removed before calculating the PSD profile)
